# Accuracy of spirometry for detection of asthma: a cross-sectional study

**DOI:** 10.1590/1516-3180.2017.0041250517

**Published:** 2017-08-21

**Authors:** Andréa Cristina Meneghini, Ana Carolina Botto Paulino, Luciano Penha Pereira, Elcio Oliveira Vianna

**Affiliations:** I MSc. Doctoral Student, Department of Social Medicine, Faculdade de Medicina de Ribeirão Preto (FMRP), Universidade de São Paulo (USP), Ribeirão Preto (SP), Brazil.; II MSc. Nurse, Department of Social Medicine, Faculdade de Medicina de Ribeirão Preto (FMRP), Universidade de São Paulo (USP), Ribeirão Preto (SP), Brazil.; III MD, MSc. Preceptor of Internship, Pneumology Service, Hospital Santa Casa de Ribeirão Preto, Ribeirão Preto (SP), Brazil.; IV MD, PhD. Associate Professor, Department of Medicine, Faculdade de Medicina de Ribeirão Preto (FMRP), Universidade de São Paulo (USP), Ribeirão Preto (SP), Brazil.

**Keywords:** Asthma, Spirometry, Methacholine chloride, Dimensional measurement accuracy, Diagnosis, Bronchial hyperreactivity

## Abstract

**BACKGROUND:**

Asthma is a chronic inflammatory disease with airway hyperresponsiveness. Spirometry is the most commonly used test among asthmatic patients. Another functional test used for diagnosing asthma is the bronchial challenge test. The aim of this study was to analyze the accuracy of spirometry for detecting asthma in the general population.

**DESIGN AND SETTING:**

Cross-sectional study with data analysis to evaluate the accuracy of spirometry through calculating sensitivity, specificity and predictive values and through the kappa agreement test.

**METHODS:**

Subjects who constituted a birth cohort were enrolled at the age of 23 to 25 years. Spirometric abnormality was defined as reduced forced expiratory volume in one second, i.e. lower than 80% of the predicted value. Measurement of bronchial responsiveness was performed by means of the bronchial challenge test with methacholine. The gold-standard diagnosis of asthma was defined as the presence of bronchial hyperresponsiveness in association with respiratory symptoms.

**RESULTS:**

Asthma was detected in 200 subjects (10.4%) out of the sample of 1922 individuals. Spirometric abnormality was detected in 208 subjects (10.9%) of the sample. The specificity of spirometric abnormality for detecting asthma was 90%, sensitivity was 23%, positive predictive value was 22%, and negative predictive value was 91%. The kappa test revealed weak agreement of 0.13 (95% confidence interval, CI: 0.07-0.19) between spirometry and the diagnosis of asthma.

**CONCLUSION:**

Spirometry, as a single test, has limitations for detecting asthma in the general population.

## INTRODUCTION

Asthma is a chronic inflammatory disease of the airways in which many cells and mediators have a role. Chronic inflammation is associated with airway hyperresponsiveness that leads to recurrent episodes of wheezing, breathlessness, chest tightness and coughing, particularly at night or early in the morning.^1^ The medical history and a physical examination are frequently sufficient for diagnosing asthma. In epidemiological studies and for screening for asthma in large groups (for example, employees at risk, students and the military), a diagnosis of asthma may be made based on questionnaires, with or without a physiological test.^2^

Spirometry is the most commonly used test for asthmatic patients. It is useful for making the diagnosis, classifying the severity and monitoring therapeutic drug administration.^3^ Use of spirometry is valued in most guidelines on approaches to and treatment of asthma.^3^^,^^4^

Another functional test that is used for diagnosing asthma is measurement of airway responsiveness, i.e. the bronchial challenge test.^5^ This test uses bronchoconstriction stimuli, such as methacholine, and it is considered to be the best choice for diagnosing asthma in patients with normal spirometry tests.^5^^,^^6^

Bronchial challenge corroborates the diagnosis of asthma through confirming the presence of bronchial hyperresponsiveness. More importantly, it may rule out this diagnosis when bronchial hyperresponsiveness is not detected, because its negative predictive value is high.^2^ For this reason, it is considered to be a gold standard test for asthma diagnosis.

Untreated asthma results in significant morbidity. It carries high costs, due not only to health expenditure but also to loss of productivity, absence from school or work and impairment of family life.^1^ One example of the importance of early asthma diagnosis is the case of work-related asthma, in which the sooner asthma is detected, the better the patient’s prognosis will be.^1^

## OBJECTIVE

The aim of this study was to analyze the accuracy of spirometry for detecting asthma in the general population, by means of calculating sensitivity and specificity and using the kappa agreement test. A positive methacholine challenge test in combination with respiratory symptoms was used as the gold-standard method for diagnosing asthma.

## METHODS

This was a cross-sectional study with data analysis to evaluate the accuracy of spirometry for detecting asthma. Spirometric abnormality was defined here as reduced forced expiratory volume in one second (FEV1), i.e. lower than 80% of the predicted value. Measurements of bronchial responsiveness were made and a gold-standard diagnosis of asthma was defined as the presence of bronchial hyperresponsiveness and respiratory symptoms.^7^

### Sample

Subjects who constituted a birth cohort were enrolled in this study at the age of 23 to 25 years. We randomly selected 2,063 subjects and 1,922 of them underwent all the procedures necessary for this study.

This birth cohort comprised subjects born in the municipality of Ribeirão Preto during the period from June 1, 1978, to May 31, 1979, and this data collection was the fourth study on this cohort.^7^

The recruitment of the sample was based on the charts of the liveborns of the original cohort, which contained the name and address of the mother and the data of birth of the child. The potential participants in this evaluation were identified by the contact and field teams, which were set up and trained over a six-month period. A period of 24 months was reserved for subject identification and for data collection, with a capacity of 4-7 daily evaluations by the service.^7^

The inclusion criteria were that the subjects needed to belong to this cohort and to be able to perform all the procedures necessary. Previous articles have reported on the history of this cohort, sample size calculations, subject selection and recruitment.^7^

This sample was assessed in the fourth phase of following up this cohort.^7^ The aim of this fourth phase was to ascertain the importance of events that occurred between the prenatal period and early adulthood and to analyze the impact of these events on the growth and development of chronic non-transmissible adult diseases, such as asthma. At this phase, anthropometric measurements were taken, blood samples were collected and spirometry, skin allergy tests and the bronchial challenge test with methacholine were performed. Asthma was one of the diseases studied. The methacholine challenge was performed on the whole sample to evaluate asthma for the several analyses on this cohort. Some of these analyses have already been published.^7^^,^^8^^,^^9^^,^^10^

The tests and measurements were carried out in a healthcare setting with easy access to medical facilities. The examinations were performed at the university hospital in the city of Ribeirão Preto, in the northwestern region of the state of São Paulo, Brazil. The participants signed a consent form after reading and listening to the aims and procedures included in the study. This study was approved by the institutional ethics committee.

### Procedures

#### Respiratory and occupational questionnaires

Respiratory symptoms were assessed using the European Community Respiratory Health Survey (ECRHS) questionnaire, as translated into Portuguese and adapted to the Brazilian lexicon.^8^ The ECRHS questionnaire was developed for use among young adults aged 20 to 44 years, to explore asthma symptoms in young adults. We used questions of the ECRHS questionnaire to explore asthma symptoms. Subjects could answer yes or no to the following questions:

- “Have you had wheezing or whistling in your chest at any time in the last 12 months?”
- “Have you woken up with a feeling of tightness in your chest at any time in the last 12 months?”
- “Have you had an attack of shortness of breath that came on during the day when you were at rest at any time in the last 12 months?”
- “Have you been woken by an attack of shortness of breath at any time in the last 12 months?”

Presence of any of these symptoms in association with bronchial hyperresponsiveness defined asthma. Thus, symptoms reported by non-hyperresponsive individuals were not enough to classify them as asthmatic.

### Spirometry and bronchial responsiveness measurements

The bronchial responsiveness to methacholine was measured using the two-minute tidal breathing method. Increasing concentrations of methacholine (0.06, 0.125, 0.25, 0.5, 1, 2, 4, 8 and 16 mg/ml) were aerosolized using a DeVilbiss 646 nebulizer (Sunrise Medical HHG Inc, Somerset, PA, USA) driven by a computer-activated dosimeter (Koko Digidoser System, PDS Instrumentation, Inc., Louisville, CO, USA) with an output of 9 ml per 0.6 second (total delivery of 0.045 ml).

FEV1 was measured at baseline and 2 minutes after each tidal breathing period. The test was stopped when either a 20% fall in FEV1 was achieved or the final concentration was reached. The challenge concentration causing a 20% fall in FEV1 (PC20) was calculated using the Koko software. We considered PC20 ≤ 4 mg/ml to indicate bronchial hyperresponsiveness. The contraindications for the methacholine challenge test were all conditions that might compromise the quality of the test or that might subject the patient to increased risk or discomfort, and these included FEV1 < 50% of predicted value, pregnancy, nursing mothers and inability to perform spirometry of acceptable quality. Other contraindications were heart attack or stroke in the last three months; uncontrolled hypertension; systolic blood pressure (BP) > 200 mmHg or diastolic BP > 100 mmHg; known aortic aneurysm; and current use of cholinesterase inhibitor medication (for myasthenia gravis). During preparation, patients were questioned about factors that could increase or decrease bronchial responsiveness, such as current respiratory infection.^2^

The hypothesis of the study was formulated after data collection. To avoid bias, the same investigator performed all tests on every volunteer. The main measurements, as described above, were symptom evaluation, FEV1 and bronchial responsiveness measurements. Based on these data, the variables were defined as follows.

### Variables

Current wheezing was defined as a positive answer to the question: “Have you had wheezing or whistling in your chest any time in the last 12 months?” Possible answers: yes or no.

Chest tightness was defined as a positive answer to the question: “Have you woken up with a feeling of tightness in your chest at any time in the last 12 months?” Possible answers: yes or no.

Breathlessness was defined as a positive answer to the question: “Have you had an attack of shortness of breath that came on during the day when you were at rest at any time in the last 12 months?” Possible answers: yes or no.

Nocturnal breathlessness was defined as a positive answer to the question: “Have you been woken by an attack of shortness of breath at any time in the last 12 months?” Possible answers: yes or no.

Reduced FEV1 was defined as any value less than 80% of the predicted FEV1. Reference values were adopted as described by Crapo et al.^11^ Thus, this quantitative variable was handled as a binary variable (FEV1 reduction: yes or no).

Presence of asthma, as confirmed through the bronchial hyperresponsiveness test, was defined by two main components of the disease, i.e. presence of bronchial hyperresponsiveness and at least one of the symptoms.^2^^,^^5^ Thus, bronchial hyperresponsiveness was handled as a binary variable (positive or negative) to define asthma.

### Sample size

The sample size was defined from previous prevalence studies in which the data gathered formed the database that allowed us to conduct the present analysis.

### Statistical analysis

Simple exploratory analyses were used to describe the study population and to calculate the prevalence of reduced FEV1, hyperresponsiveness, respiratory symptoms and asthma, expressed as percentages. 

We first tested the association between reduced FEV1 (under test here) and asthma as confirmed through the methacholine challenge test (gold standard). If there was no association, we would not pursue this to test its accuracy.

The simple and multiple log-binomial regression method was used to estimate the prevalence ratio, since the response was binary (presence of disease = yes or no). In assessing the association between reduced FEV1 and asthma, the confounding variables were schooling, type of work, smoking, physical activity, blood pressure, allergy and anthropometry.

The presence of bronchial hyperresponsiveness (i.e. a positive result from the methacholine challenge) in subjects with symptoms was the definition used for asthma (gold standard). The accuracy of FEV1 reduction for detecting asthma was tested through calculation of positive and negative likelihood ratios and sensitivity and specificity. The kappa test was applied to check for agreement between reduced FEV1 and asthma. The analyses were carried out using STATA version 9.1 (Copyright 1984-2005; Stata Corp., 4905 Lakeway Drive, College Station, Texas 77845, USA).

## RESULTS

The age of the sample of 1922 individuals (980 women) who completed the protocol (mean ± standard deviation, SD) was 23.9 ± 0.7 years. The prevalence of asthma was 10.4% and 119 subjects (6.1%) were using asthma medications. The prevalences of the major variables are shown in Table 1. Bronchial hyperresponsiveness was detected in 22.2% of the sample, i.e. 427 subjects: 263 women (61.6%) and 164 men (38.4%) (P < 0.0001). Current smoking was reported by 17.4% of the individuals, i.e. by 14.2% of the women and 20.7% of the men (P < 0.0001). The prevalence of current plus former smoking was 26.3%, i.e. 21.9% of the women and 30.8% of the men (P < 0.0001). Slightly more than one third of the individuals belonged to the categories of qualified and semi-qualified manual workers; 21.5% to the unqualified manual category; 21.1% to the non-manual category; and 22.7% did not belong to the economically active population. Regarding educational background, 14.7% had had 1-8 years of schooling, 50.9% had had 9-11 years and 34.4% had had more than 11 years.

**Table 1:** Prevalence of reduced FEV1, hyperresponsiveness, respiratory symptoms and asthma

Variables	Total		Males (n = 942)		Females (n = 980)	
	n	%	n	%	n	%
Wheezing	366	19.05	159	16.90	207	21.12
Chest tightness	231	12.04	65	6.91	166	16.96
Shortness of breath at rest	229	11.92	61	6.48	168	17.14
Breathlessness	169	8.80	49	5.21	120	12.24
PC20 ≤ 4 mg/ml	427	22.22	164	17.41	263	26.84
Reduced FEV1	208	10.88	88	9.41	120	12.30
Asthma	200	10.40	67	7.10	133	13.60

FEV1 = forced expiratory volume in one second; Reduced FEV1 means values below 80% of predicted value; PC20 = challenge concentration causing 20% decrease in FEV1; PC20 ≤ 4 mg/ml = bronchial hyperresponsiveness.

In the 208 cases with FEV1 reduction, the FEV1/FVC ratio (% of predicted value) was 82 ± 6% (mean ± SD). Since 90% of the predicted value is considered to be the cutoff value for this variable, these findings indicate that there was a reduction in FEV1/FVC in the group with FEV1 reduction. The prevalence of FEV1/FVC reduction in this group was 70%. FEV1 (% of predicted value) for the group with reduced FEV1 (n = 208) was 73 ± 0.06%.

The association between asthma and reduced FEV1 is shown in Table 2. The kappa index calculated to assess the agreement between reduced FEV1 and asthma was 0.13 (range, 0.07 - 0.19), thus indicating weak agreement. The sensitivity, specificity, predictive values, positive likelihood ratio and negative likelihood ratio for reduced FEV1 to detect asthma were 23%, 90%, 22%, 91%, 2.30 and 0.86, respectively (Table 3). The prevalences of reduced FEV1 in cases of confirmed asthma and in cases of non-asthma are shown in Table 4.

**Table 2:** Univariate and multivariate binomial analysis on reduced FEV1

	Univariate analysis	Multivariate analysis
	PR (95% CI)	PR (95% CI)
Asthmatic	2.45 (1.83-3.29)	2.08 (1.52-2.84)
Level of schooling 1	0.70 (0.34-1.42)	0.82 (0.39-1.75)
Level of schooling 2	0.62 (0.32-1.19)	0.79 (0.39-1.61)
Level of schooling 3	0.41 (0.21-0.81)	0.70 (0.33-1.49)
Skilled crafts	1.20 (0.70-2.03)	1.22 (0.69-2.15)
Semi-skilled manual work	2.13 (1.34-3.36)	1.87 (1.12-3.12)
Unskilled manual work	2.01 (1.33-3.03)	1.75 (1.09-2.80)
Smoking	1.09 (0.78-1.51)	1.07 (0.76-1.49)
Regular physical activity	1.45 (1.12-1.88)	1.30 (1.00-1.69)
Waist-to-height ratio	1.04 (0.79-1.37)	1.07 (0.81-1.42)
Arterial blood pressure	0.71 (0.50-1.00)	0.76 (0.53-1.09)
Allergy	1.09 (0.84-1.42)	1.05 (0.80-1.38)

FEV1 = forced expiratory volume in one second; PR = prevalence ratio; CI = confidence interval; level of schooling 1 = 9-11 years of schooling; level 2 = 5-8 years of schooling; level 3 = 4 years of schooling or less. FEV1 reduction means values below 80% of predicted value.

**Table 3:** Sensitivity, specificity, predictive values and likelihood ratio values for reduced FEV1 to detect asthma

Parameter	Value
Sensitivity	23%
Specificity	90%
Positive predictive value	22%
Negative predictive value	91%
Positive likelihood ratio	2.30
Negative likelihood ratio	0.86

FEV1 = forced expiratory volume in one second; reduced FEV1 means values < 80% of predicted value. Asthma was defined as positive methacholine bronchial challenge test in association with any compatible symptom.

**Table 4:** Reduced FEV1 in cases of confirmed asthma and in cases of non-asthma

	Confirmed asthma	Non-asthma	Total
	n = 200	n = 1,722	1,922
Reduced FEV1	46	162	208
Normal FEV1	154	1,560	1,714

FEV1 = forced expiratory volume in one second; Reduced FEV1 means values below 80% of predicted value; Asthma was defined as positive methacholine bronchial challenge test in association with any compatible symptom.

## DISCUSSION

**Figure 1:**
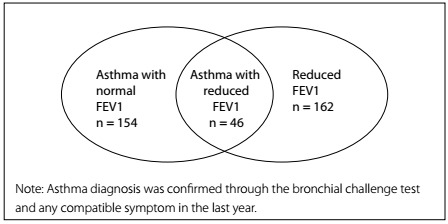
Number of subjects with asthma and with reduced forced expiratory volume in one second (FEV1).

The aim of this study was to analyze the accuracy of spirometry for detection of asthma in general or specific populations. Accuracy was evaluated by means of calculation of sensitivity and specificity and use of the kappa agreement test. A positive methacholine challenge test in combination with asthma symptoms was used as the gold-standard reference method for diagnosing asthma. A preliminary statistical analysis was conducted to investigate the association between abnormal spirometry and asthma in this sample. This first step confirmed that there was an association between reduced FEV1 and asthma. However, subsequent analyses indicated that spirometry underdiagnosed asthma. The sensitivity of spirometry was 23%, specificity was 90%, negative predictive value was 91% and positive predictive value was 22%. The accuracy calculated using the Youden index was low (0.33). The agreement between asthma and reduced FEV1 was also low according to the kappa coefficient (0.13). Figure 1 shows the data relating to this lack of agreement.

This high negative predictive value for spirometry (91%) means that a negative test (normal spirometry) probably indicates absence of asthma. It is worthy bearing in mind that predictive value calculations are influenced by prevalence, i.e. the low probability of asthma in this case of a negative test was also a consequence of the low prevalence of asthma (10.4%). The low sensitivity of this test indicates that it is not suitable for disease screening. Although spirometry is a cheap, common and simple health screening test that, for example, can be used for workers exposed to high-risk pollutants, it is insufficient for detecting most cases of asthma.^12^

In the first study evaluating the diagnostic accuracy of spirometry for diagnosing airflow obstruction in patients with asthma or chronic obstructive pulmonary disease (COPD) in primary care, it was found that the presence or absence of COPD could be estimated with comparatively high diagnostic accuracy. It was also possible to accept the presence of asthma. However, it was impossible to rule out asthma because the sensitivity was too low. The sensitivity for diagnosing airway obstruction in asthma cases was 29% (95% confidence interval, CI: 21-39) and the specificity was 90% (95% CI: 81-95).^13^ These figures were similar to our findings.

Four commonly used tests for diagnosing asthma were assessed in a population-based sample of 495 schoolchildren (the Odense Schoolchild Study). The test panel consisted of FEV1, challenge with treadmill exercise, challenge with inhaled methacholine and monitoring of peak expiratory flow (PEF), twice daily for two weeks. The agreement between the four tests was weak. The sensitivity for diagnosed asthma was highest for the methacholine challenge followed by PEF monitoring, whereas the specificity for asthma or asthma-like symptoms was marginally higher with the other two tests (spirometry and challenge with exercise).^14^ Finally, Bonini et al. reported that spirometry appeared to be poorly sensitive for detecting mild persistent or intermittent asthma in athletes, possibly because exercise necessities are associated with spirometry values of approximately 100% of predicted values in most athletes.^15^

Most definitions of asthma highlight variable airflow obstruction and inflammation as essential elements of this condition. These characteristics do not translate into an unambiguous definition to separate asthma and non-asthma in surveys and in screening approaches. In previous studies from our group, we showed that non-dichotomous definitions of asthma may lead to different results regarding the prevalence of and risk factors for asthma.^16^ Nevertheless, asthma definitions depend on the purposes of the diagnosis and definitions based on spirometry are scarcely found in epidemiological studies.^16^^,^^17^

Conversely, for medical practice, use of spirometry is certainly more important because patients have asthma of greater severity and present with bronchial obstruction. Measurement of FEV1 is necessary for classifying its severity and for follow-up. In cases with normal spirometry and unconfirmed asthma, bronchial challenge with methacholine is the test of choice.^2^

Work-related asthma is the most prevalent occupational respiratory disease. It is defined as asthma that is causally and specifically related to exposure to airborne dust, gases, vapors or fumes in the workplace. Work-related asthma encompasses aggravated asthma (meaning preexisting asthma) and occupational asthma (without preexisting asthma).^2^ Patients’ prognoses depend on the duration of exposure, duration of symptoms and severity of asthma at the time of diagnosis. Patients with asthma of greater severity tend to continue to present asthma symptoms after exposure ceases, but those with mild asthma may subsequently achieve complete remission.^2^ Therefore, early diagnosing of asthma and exposure cessation are the most important approaches for work-related asthma.^1^

Brazilian regulation NR-7 requires annual spirometry for workers dealing with non-fibrogenic aerosols. Our finding that spirometry has low sensitivity for detecting asthma in the general population does not support this policy. Instead, regulations should encourage use of the bronchial challenge test and symptom questionnaires to detect asthma cases with normal spirometry. Other manifestations that precede asthma could also improve patients’ odds of early detection and cure for occupational asthma. These may include occurrences of rhinitis, skin symptoms and allergic sensitization. Therefore, NR-7 may lead to detection of asthma at a later stage than would be recommendable.^12^

However, the purpose of the present analysis was not to examine the efficacy of the regulation NR-7. We mention it simply as an example of the value that is placed on spirometry, which deserves attention. If NR-7 were to be changed such that it would then recommend use of the bronchial challenge test or use of a symptom questionnaire, this would lead to earlier diagnosis of asthma in workers with high-risk exposure.

Bronchial hyperresponsiveness is the best test for detecting asthma, but this procedure carries risks and precautions are required. Occasional dramatic falls in FEV1 may occur during testing and the risk of such events may be higher in individuals with low baseline lung function. The hazards and reactions include bronchoconstriction, hyperinflation with severe coughing, dizziness, lightheadedness or chest pain. Nurses or technicians who have asthma should not administer methacholine. It is difficult to implement this procedure because of the safety measures that are necessary and the costs of this procedure. Typically, the methacholine test is performed in a pulmonary function laboratory, a clinic or a physician’s office. Prices in the United States may be estimated from a review article published by Sam Birnbaum and Timothy J. Barreiro in 2007: “The Centers for Medicare and Medicaid Services reimbursement for methacholine challenge test is approximately $175. However, reimbursement from commercial insurers may vary dramatically”.^18^

The positive features of the present study protocol were its large sample size, its excellent reference test for asthma and the young age of the participants, which practically excluded the bias of patients with COPD. One limitation of the study was its lack of diagnosis for all subjects with reduced FEV1. Asthmatics could be identified through the methacholine challenge, but other diseases with normal bronchial responsiveness were not evaluated. However, detection of reduced FEV1 was not common. Subjects without asthma but with reduced FEV1 may have obesity, chest wall disease, neuromuscular impairment, bronchiectasis or other respiratory diseases.

## CONCLUSION

The low sensitivity of spirometry for detecting asthma in clinical settings allows us to assume that this test conducted alone is not a good screening tool.
